# A collaboration team to build social service partnerships within a safety-net health system

**DOI:** 10.1186/s12889-024-18155-z

**Published:** 2024-03-01

**Authors:** Savanna L. Carson, Francesca Cameron, Diamond Lee, Diana Zúñiga, Kelli Poole, Adjoa Jones, Cristina Herman, Mayra Ramirez, Simone Harlow, Jeannette Johnson, Etsemaye P. Agonafer, Clemens S. Hong, Arleen F. Brown

**Affiliations:** 1 Division of General Internal Medicine and Health Services Research, UCLA David Geffen School of Medicine, 1100 Glendon Ave, Suite 1100, Los Angeles, CA 90095 USA; 2 Los Angeles County Department of Health Services, 711 Del Amo Blvd, Torrance, CA 90502 USA; 3 Tres Lunas Consulting, 1509 Stanley Ave. #302, Long Beach, 90804 USA; 4 Los Angeles County Department of Health Services, 313 N Figueroa St, Los Angeles, CA 90012 USA; 5 Department of Health System Science, Kaiser Permanente Bernard J. Tyson School of Medicine, Pasadena, CA USA

**Keywords:** Safety net, Community engagement, Health and social care integration, Health disparities, Social determinants of health

## Abstract

**Background:**

To facilitate safety-net healthcare system partnerships with community social service providers, the Los Angeles County Department of Health Services (LAC DHS) created a new collaboration team to spur cross-agency social and medical referral networks and engage communities affected by health disparities as part of a Sect. 1115 Medicaid waiver in Los Angeles County entitled Whole Person Care-Los Angeles (WPC-LA).

**Methods:**

This observational research reviews three years of collaboration team implementation (2018–2020) through Medicaid-reportable engagement reports, a collaboration team qualitative survey on challenges, facilitators, and recommendations for community engagement. Member reflections for survey findings were conducted with the collaboration team and LAC DHS WPC-LA leadership.

**Results:**

Collaboration team Medicaid engagement reports (*n* = 144) reported > 2,700 events, reaching > 70,000 individuals through cross-agency and community-partnered meetings. The collaboration team survey (*n* = 9) and member reflection sessions portrayed engagement processes through outreach, service assessments, and facilitation of service partnerships. The collaboration team facilitated community engagement processes through countywide workgroups on justice-system diversion and African American infant and maternal health. Recommendations for future safety net health system engagement processes included assessing health system readiness for community engagement and identifying strategies to build mutually beneficial social service partnerships.

**Conclusions:**

A dedicated collaboration team allowed for bi-directional knowledge exchange between county services, populations with lived experience, and social services, identifying service gaps and recommendations. Engagement with communities affected by health disparities resulted in health system policy recommendations and changes.

## Introduction

California and other states with expanded Medicaid coverage are re-envisioning health and social service partnerships to address unmet social needs and reduce health disparities and costly acute healthcare utilization [1–4]. Improving health equity requires social care coordination between health systems, social service agencies, and community resources addressing the social determinants of health [5–7]. Policy shifts integrating social care coordination into healthcare delivery have helped initiate community partnerships or community stakeholder committees, facilitated service coordination, developed interventions for quality of care, and provided insight into local needs, resources, and strategies for culturally congruent care [8, 9]. In recent years, Sect. 1115 Medicaid waivers funded initiatives coordinating social care into healthcare delivery, including building or strengthening infrastructure for community partnerships for social needs services [10]. In California, 25 programs in 26 counties implemented the Medicaid (called Medi-Cal in California) 1115(a) waiver “Whole Person Care” (WPC) pilot to integrate the social and medical needs of high-risk, high-utilizing Medi-Cal beneficiaries [10]. Medi-Cal WPC Pilots were required to improve care coordination, access to care, and integrate services among “local entities that serve the target population” [11]. The limited statewide funding waiver was funded from 1/1/2016–12/31/2020 but was extended through December 2021, and lessons learned have since been incorporated within California Advancing and Innovating Medi-Cal (CalAIM) which leverages Medicaid to achieve a socially integrated health delivery system, promoting health equity by addressing the complex clinical and social needs facing patients with high utilization of acute care services [12].

The Los Angeles County Department of Health Services (LAC DHS) Medicaid WPC pilot was entitled Whole Person Care-Los Angeles (WPC-LA). WPC-LA aimed to deliver coordinated services to the most vulnerable Medicaid beneficiaries [10, 13]. WPC-LA integrated health, behavioral health, and social service systems through a community health worker (CHW) model [14] for six high-risk populations experiencing homelessness, justice involvement, barriers to a healthy pregnancy, mental health disorders, substance use disorder, and complex health conditions [13]. While LAC DHS is the primary administrator of WPC-LA, the Los Angeles County (LAC) Departments of Public Health (LAC DPH), Mental Health (LAC DMH), Public Social Services (DPSS), and Justice Departments (Sheriff’s office, Office of Diversion and Reentry, Probation, etc.) are key county stakeholders in addition to health plans, clinics, and community-based organizations (CBOs). CHWs, social workers, case managers, and other direct service professionals promoted health and social care continuity, connecting WPC-LA patients to primary care, specialty care, and social services, including housing, substance use treatment, reentry services, employment, food, and legal advocacy.

To support coordinating social services in healthcare delivery, WPC-LA developed a new collaboration team in the LAC DHS health system for community engagement, partnership, and cross-agency collaboration to support the six populations' tailored health and social needs. The goals of the team included: (a) initiate and foster purposeful engagement to increase awareness of WPC-LA program offerings and expand the social service referral network; (b) identify critical assets and gaps within the county’s health and social services with a focus on reducing health disparities; (c) build community capacity to promote health equity through collaborations addressing social determinants of health for WPC-LA populations, and (d) create lasting pathways for diverse community voices in the decision-making process through community action teams and workgroups dedicated to problem-solving within health inequities. Herein is a descriptive study that describes the roles of this novel health-system-embedded collaboration team and challenges, facilitators, case studies, and recommendations for health-system community engagement processes for addressing health disparities, service gaps, and social needs.

## Methods

UCLA researchers partnered with the LAC DHS collaboration team to conduct narrative and observational research documenting lessons learned and recommendations for creating safety-net health system collaboration with local communities on social service partnerships and health disparities. This study was found to be exempt by the UCLA Institutional Review Board. We report our findings using the Standards for Reporting Qualitative Research [15, 16].

### Setting and role of the collaboration team

**Fig. 1:**
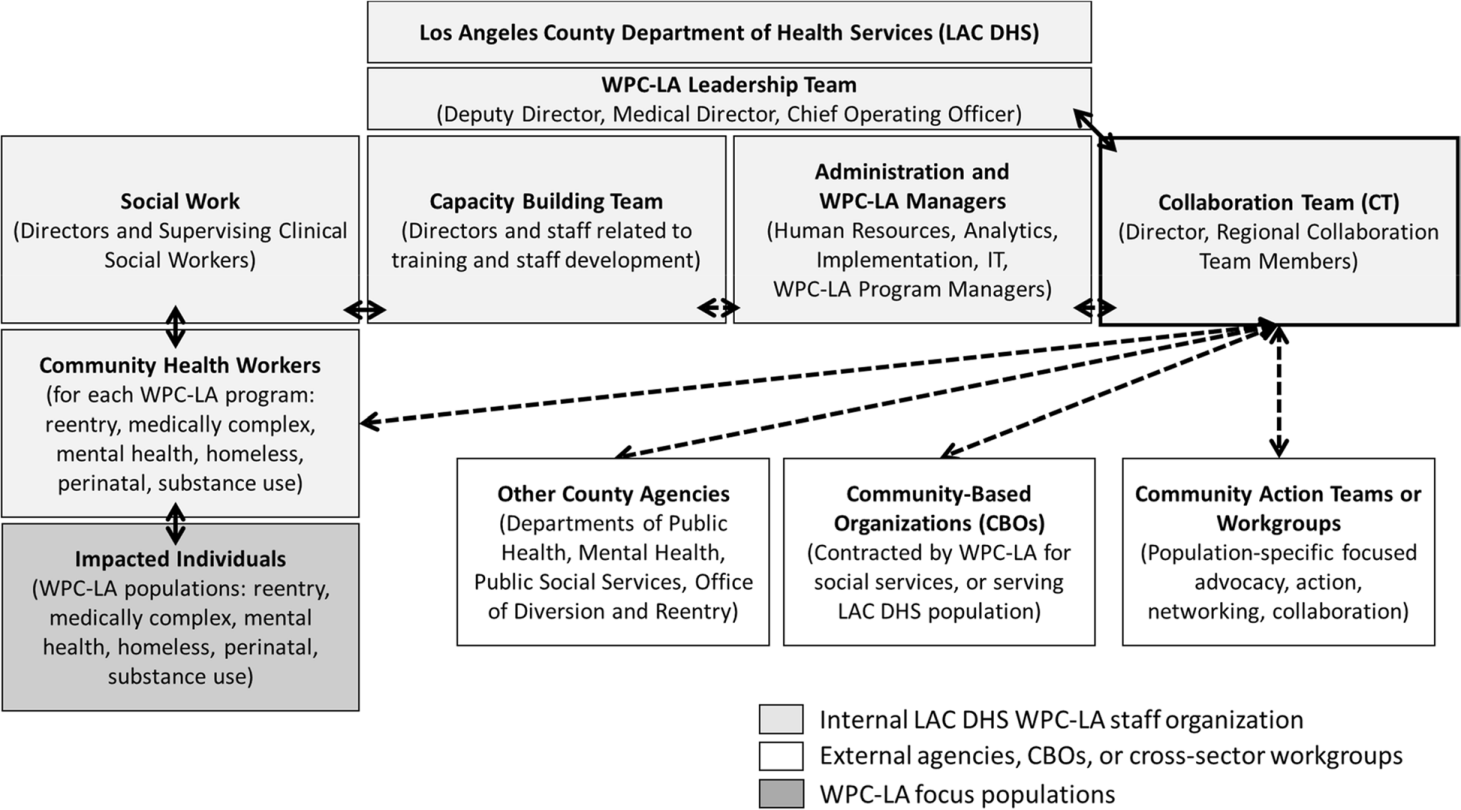
LAC-DHS Whole Person Care-Los Angeles staff structure* *This figure focuses specifically on highlighting the Collaboration Team’s structure within the health system and does not comprehensively display levels of interaction between the other roles, positions, or agencies. For instance, CHWs conducted referrals to CBOs, whereas the Collaboration Team conducted outreach and partnership on agency-wide levels

Collaboration team members (*n* = 9) were new LAC DHS hires between 9/2017 and 3/2018. Recruitment focused on individuals with experience in nonprofits, social services, and grassroots community organizing and lived experience or shared adversity related to the six WPC-LA focus populations. Collaboration team members were full-time LAC DHS employees who directly reported to WPC-LA leadership. As a health system representative, each collaboration team member worked across eight Los Angeles County geographic regions called Service Planning Areas (SPAs). The role facilitated regional service delivery, efficiency, and collaboration across the WPC-LA structure (from CHWs to WPC-LA leadership), government agencies providing public health, social services, mental health, and justice care (including LAC DPH, DMH, DPSS, and City or County Justice Systems), and CBOs providing social services (homeless shelters, substance use treatment, legal aid, etc.). The structural organization of the collaboration team within LAC DHS’s WPC-LA leaders, government agencies, and community entities is depicted in Fig. 1. Herein, the term collaboration within “collaboration team” refers to the three components described by Berkowitz [17], relating to the collective effort, a social change process, and information exchange for mutual benefit.

### Data collection and analysis

Following three years of collaboration team implementation within WPC-LA (9/2017–12/2020), UCLA researchers used the CDC’s six-step framework process for organizing program evaluation [18] to: a) compile and analyze LAC DHS reports on the collaboration team’s collaboration activities for Medi-Cal reporting, b) conduct a qualitative LAC DHS collaboration team survey on challenges, facilitators, and recommendations for the health-system community engagement, and c) hold member reflection meetings to refine survey findings with the collaboration team and LAC-DHS WPC-LA leadership.

- *Medi-Cal Collaboration reports*. LAC DHS monthly and annual engagement activity reports were compiled from 1/2018–12/2019, encompassing the number of events, attendees, and event focus population (homelessness, justice involvement, barriers to a healthy pregnancy, mental health disorders, substance use disorder, and complex health conditions, or other). These reports were generated quarterly by LAC DHS and sent to the State of California Health and Human Services Agency to determine stakeholder engagement and outreach, Medi-Cal resource use, and receive Medi-Cal incentives, per Medi-Cal waiver requirements for improving the integration of care coordination among county agencies and appropriate care services [11]. Engagement events included meetings with non-LAC-DHS collaborators working on service integration and collaboration, including community members, CBOs, or county agency stakeholders, i.e., DMH or DPSS. Reports following the onset of the COVID-19 pandemic in March 2020 were not counted herein as collaboration team efforts shifted to meet pandemic needs. Collaboration outcome reports are tallied herein to exemplify cross-sector collaboration connections conducted by the collaboration team.
- *Collaboration Team survey*. A survey was distributed to the nine original collaboration team members in February and March 2020 and solicited demographic and open-ended questions on successes, challenges, facilitators, and recommendations for other safety net systems for community engagement processes. See Table 1 for survey questions. Qualitative results from the Collaboration Team survey were analyzed using reflexive thematic analysis by an experienced qualitative researcher [SC] unaffiliated with LAC DHS and research findings [19, 20].
- *Member reflections*. Following analysis, we reported and reviewed preliminary themes in eleven subsequent 1–2 h meetings with the Collaboration Team (*n* = 7 meetings, 9 Collaboration Team members) and LAC-DHS leadership (*n* = 4 meetings with three individuals). The meetings aimed to obtain member reflections on preliminary qualitative analysis, where participants are invited to provide further insight and understanding of findings [21, 22]. These member reflection meetings included presenting a subset of preliminary themes from the survey, inviting participants to provide descriptive examples of case studies representing the themes, or providing comments, reflections, or suggested edits for the preliminary results. Meeting notes were utilized to validate, refine, and expand themes. These sessions allowed for probing how collaboration team outreach, partnerships, and service assessments were conducted and obtaining recommendations for future social service and safety-net collaboration.

**Table 1:** Collaboration team survey questions

1. Race/Ethnicity
**Characteristics of an Effective Regional Collaboration Team**
1. Briefly, how do you define collaboration and community engagement in your role?
2. What attributes affect collaboration and community engagement (pre-existing connections to community, lived experience, etc.)? How do these attributes facilitate or hinder your work?
3. What does community power-sharing look like in your work? Please give concrete examples 4. How do team-building activities impact your ability to do your work?
5. How do team-building activities impact your well-being?
**Community Engagement Impacts**
6. How does your work support frontline providers (i.e., CHWs, Patient Navigators, street-outreach teams, etc.)?
7. What impact has the work of the Collaboration Team had on community organizations?
8. What impact has the work of the Collaboration Team had on healthcare leaders?
9. What impact has the work of the Collaboration Team had on policymakers?
**Community Engagement Challenges/Facilitators**
10. What were the three main factors that helped you to do your work effectively?
11. What three main factors made it difficult to do your work effectively?
**Recommendations:** *For these questions, imagine another safety net system that plans to create its own Collaboration Team to engage more effectively with communities*
12. What advice would you give another health system on developing its own Collaboration Team?
13. How can this role evolve further in the health system?
14. How should health systems prepare to effectively engage with the communities they serve?
15. What strategies should be put in place to help health systems become more responsive to input from the community?

## Results

### Demographic characteristics of the collaboration team

The nine WPC-LA collaboration team members active at the time of this evaluation were all women, identifying as Black/African American (3), biracial (2), Latina (2; one of Central American descent, and another of Mexican/Chicana descent, both identified as Indigenous), White (1), and Asian American (1). Types of lived experience collaboration team members portrayed reflected WPC-LA focus populations, including personal or familial experience with incarceration, substance use, barriers to reproductive healthcare, mental health, complex health conditions, homelessness, racism, and use of public benefits.

### Medi-Cal collaboration reports

From the LAC-DHS Medi-Cal collaboration reports (*n* = 144), we identified over 2,700 in-person collaboration events reaching over 70,000 people from January 2018 to March 2020 to increase awareness of WPC-LA programs and expand the social service referral network. Outreach ranged from 10 to 60 meetings per month per Collaboration Team member. A description of outreach activities is described next.

### Survey and member reflection results

**Fig. 2:**
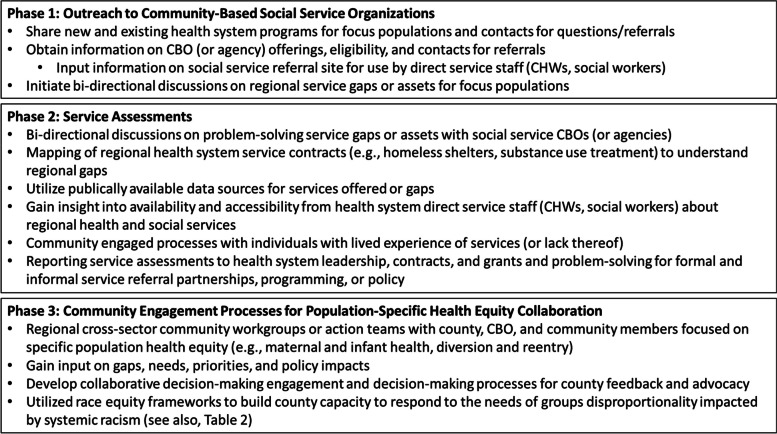
Collaboration team community engagement processes

The survey results (*n* = 9 respondents) and Collaboration Team and LAC DHS leadership member reflections (*n* = 11 meetings with 9 Collaboration Team members and three LAC DHS health system leadership individuals) describe the collaboration team's evolutionary, phased role in outreach, service assessments, and building engagement processes, (see Fig. 2), assets and challenges faced during implementation, and recommendations and lessons learned for other safety net systems.

### Phase 1: collaboration team outreach: initiating health system relationships with local community social service organizations

The collaboration team worked in specified Los Angeles SPAs to conduct outreach meetings with local direct-service CBOs and county agencies, including DPSS, LAC DMH, and LAC DPH, on services related to WPC-LA focus populations. The purpose was to (1) increase local knowledge of WPC-LA program offerings for LAC DHS referral, (2) build a bilateral social service referral network for CBOs, county agencies, and LAC DHS WPC-LA staff, such as CHWs or case managers, and (3) initiate bi-directional discussions on service gaps or assets for the WPC-LA focus populations. Outreach focused on social services serving LAC-DHS focus populations, such as regional LAC DMH health neighborhood groups, local homeless coalitions or agencies, legal aid organizations, health services, reentry services, and maternal health advocacy groups. Outreach continued using snowball sampling and referrals from the county or community contacts. Outreach meetings included a general WPC-LA overview, WPC-LA program services and eligibility for the six WPC-LA focus populations, referral information, and discussion of any congruent services or service gaps. Meetings would attempt to reach multiple staff levels within CBOs (CHWs to CBO directors). The in-person outreach supported knowledge sharing of available services and care coordination processes, built relationships with local agencies and community stakeholders, and provided direct bilateral contacts at each entity. As a collaboration team member explains,

“*Collaboration is a process and an outcome. As a process, it takes building authentic and supportive relationships with individuals and organizations. As an outcome, it creates strong partnerships and programs. Community engagement in our role is also about building genuine, supportive, mutually beneficial relationships."*

The team collected public-facing information on CBO services from outreach meetings, including services, hours, and eligibility. Information was added to OneDegree (1degree.org), an online referral platform for local social, health, and other services (i.e., employment, public benefits, and food pantries). WPC-LA collaboration team members and CHWs added over 1,000 CBOs from 2019 to 2020 and created multiple specialty social and medical resource websites tailored for WPC-LA population-specific needs (i.e., reentry, housing, reproductive health) on OneDegree.

### Phase 2: collaboration team service assessment: identifying inequitable assets and gaps of health and social services through community engagement

After outreach presentations, collaboration team members led discussions on available regional services for the six WPC-LA focus populations. For instance, if a CBO described support services for low-income mothers, discussions could evolve to describe how WPC-LA initiatives (e.g., doulas) may support their clients and vice versa. Or, if discussions revealed a lack of particular services in their region, the team would compile and report service gaps to WPC-LA leadership. The collaboration team mapped WPC-LA service contracts by SPAs to illustrate region-specific service gaps (e.g., homeless shelters, substance use treatment centers, or reentry services). Additional supportive data sources for service assessments included publicly available data from county agencies, shared lived experiences from focus populations, or feedback from WPC-LA CHWs on local resource availability and accessibility (i.e., limited hours or eligibility). Regional assessments helped identify potential interventions, brought health system staff to community spaces, and elevated community-identified service needs to health system leaders.

Following service assessments, the collaboration team would meet with the LAC DHS leadership and contracts and grants division to strategize improving WPC-LA service partnerships, including creating new grant proposals and contracts or expanding existing contracts for additional services. When specific WPC-LA resources or contracts were unfeasible, the team focused on developing partnership strategies for service gap problem-solving, such as creating a Memorandum of Understanding. For instance, a Memorandum of Understanding between LAC DHS, Los Angeles City jails, and local providers was signed to allow WPC-LA CHWs to refer clients to an existing City Jail Diversion program directly. Another example includes a Memorandum of Understanding between LAC DHS and LAC DPH to refer LAC DHS clients to the LAC DPH Doula program that allows for Doulas in LAC DHS hospitals. The collaboration team's ability to reach direct service staff (i.e., CHWs) and LAC-DHS leadership (including contracts administration) helped support cross-sector, multi-level problem-solving. A collaboration team member describes how focused discussions across the county, including within county services, were novel,

"*A success of the WPC pilot on regional cooperation is just getting to a community and identifying the gaps in services and resources, networking and bringing community leader[s] and members, and local politicians involved in these conversations… [it is] another example of what the community, local government, local public health departments, [and] mental health departments can do to affect change*."

### Phase 3: collaboration team community engagement processes: bridging health and social system collaboration for health equity

The team organized several place-based initiatives to build cross-sector and community-focused capacity to improve equitable services. Case studies describe examples of building new health systems and community engagement processes through countywide action teams and workgroups on justice-system diversion and African American infant and maternal health (see Table 2). As building community engagement processes for health systems within social and medical integration is new, these case studies portray collaboration team community engagement strategies for gaining cross-sector input in health programs and policies for specific populations facing health disparities.

**Table 2:** Building health system engagement case studies

**(1) Los Angeles County Alternatives to Incarceration Workgroup**
– **Goal**: Develop a roadmap of policy strategies to scale alternatives to incarceration and diversion through preventative health and social care
– **Health Disparity**: Before the COVID-19 pandemic, of the county’s ten million residents, 74% of arrestees were Black and Latinx. While only 9% of the county residents are Black and 49% are Latino, they comprise 29% and 52% of the jail population, respectively [58].
– **Collaboration Team Role**: Co-facilitators, organizers
– **Partners**: LAC DHS, LAC DPH, and other community and government stakeholders [58]. A Los Angeles County Board of Supervisors February 12, 2019 motion aided the official founding and funding
– **Community engagement and power-sharing strategies**: Developed six ad hoc committees compromising broad expertise (Community-Based System of Care, Community Engagement, Data, Research, Racial Equity, Funding, Gender, and Sexual Orientation, Justice System Reform) with voting privileges shared between government and community leaders
– **Outcomes**:
o 70 public consensus-building meetings from 9/2019 to 3/2020
o Meta-analysis of California policies for reducing incarceration (e.g., career services, reentry support, housing, legal aid, etc.) and diversion processes (e.g., collaborative community courts, specialized supportive housing programs, mental health full-service partnerships, and sobering centers) [59].
o County reentry resources were compiled for a One Degree ommunity resource page [60].
o Published the *Care First, Jails Last: Health and Racial Justice Strategies for Safer Communities *[58] report, including five strategies and 114 recommendations to expand services and promote continuous community engagement and consensus-building to inform government policy decisions. On 3/10/2020, the county Board of Supervisors adopted all five reported strategies
o The workgroup has served on multiple countywide public policy initiatives, including the engagement plan to close LAC’s Men’s Central Jail [61],, the development of an alternative crisis response system [62], and Measure J development [63]; a county ballot initiative passed on 11/3/2020 redirecting 10% of net county costs towards incarceration alternatives and housing
**(2) African American Infant and Maternal Mortality Prevention Initiative Community Action Team**
– **Goal**: Address the unacceptably high rates of Black infant and maternal deaths countywide and ensure healthy and joyous births for Black families in LA County
– **Health Disparity**: Though local, state, and federal initiatives provide funds and programs to address African American maternal and infant health outcomes, such as doula programs, local community voices most impacted by perinatal disparities were often missing from decision-making spaces. County leadership also lacked African American representation in leadership positions. In 2017, the LA County Health Agency (LAC DHS, LAC DPH, and LAC DMH) reported racism as a leading cause of Black infant deaths setting a 5-year goal to reduce the black-white infant mortality gap by 30% [64]. – **Collaboration Team Role**: Co-facilitators, organizers
– **Partners**: LAC DHS, LAC DMH, LAC DPH, First 5 LA, community organizations, mental and health care providers, funders, and community members
– **Community engagement and power sharing strategies**: Developed five place-based cross-sector working groups and regional working groups focused on local resources for mothers and parents
– **Outcomes**:
o From 11/2018 to 4/2020 the initiative led over 60 meetings focused on maternal and infant health equity with participation from over 500 community members and partners
o The initiative worked to develop the “*Birthing People’s Bill of Rights*,” in July 2020 providing education for birthing moms during COVID-19 when doulas or family were not allowed in hospitals[65]. The initiative distributed the bill of rights to partner CBOs, birthing mothers, and regional hospitals
o Targeted weekly newsletters with local resources for pregnancy and parenting to over 500 community stakeholders (e.g., breastfeeding week events, parenting resources, and food giveaways)

Initiatives emphasized power-sharing by obtaining community expertise, gaining insight from individuals with lived experience, informing program decision-making, guiding local policy initiatives, and building cross-sector partnerships to address social service gaps. Inclusive engagement process practices included lay-level communications, equal voting privileges for community stakeholders, consensus-building processes, transparent decision-making, and elevating the voice of those with lived experience. A team member explains the strategic inclusion of WPC-LA's target populations as "*constantly engaging individuals directly impacted by an issue to participate in a community or government initiative*."

For example, one collaboration team member [D.L.] described regional coordination of homeless agencies and hospital stakeholders to conduct strategic planning for the 2019 California SB-1152 Hospital patient discharge planning bill, requiring a hospital to document discharge planning (including shelter or social services) before discharging a homeless patient. The collaboration team developed a working group in their SPA to educate, share information, identify resources, and coordinate planning between local health systems and CBOs.

Another member [D.Z.] described a process of engaging justice systems, justice-involved CBOs, and community members in advocating for incarcerated person's ability to identify family members' disclosure of their medical records. Family disclosure is allowed through the HIPAA Privacy Rule and can promote post-release healthcare continuity and support community reentry. However, it was unavailable through LAC-DHS correctional health. Advocacy was conducted with LAC-DHS WPC-LA leadership, eventually leading to medical record functionality for adding family member HIPAA disclosure in May 2021.

### Facilitators identified by the collaboration team in conducting health system community engagement

Collaboration team members noted their prior community expertise, relationships, and lived experience as related to WPC-LA focus populations as a strength in working within promoting cross-sector partnerships. Team members described community representation as an asset for engagement, mutual understanding, and trust-building with community members and CBOs. Members described strengths in internal collaboration team activities for grounding community engagement through weekly team-building and bi-annual retreats. These activities included staff development, wellbeing workshops, discussions on lessons learned from new initiatives, and the development of internal frameworks and strategic plans, including a racial equity and social justice plan. Tools, readings, and frameworks used for team building sessions included community engagement principles [23, 24], human-centered design [25], social change and dismantling racism tools [26, 27], racial identity development readings [28], and various publicly available tools from the Government Alliance on Race and Equity [29–34] and Racial Equity Tools websites [35].

### Challenges identified by the collaboration team in conducting health system community engagement

Challenges were noted in the initial outreach phase to social service CBOs. Due to the rapid WPC-LA ramp-up for pilot Medicaid funding, where WPC-LA staffing and services varied by SPA in the initial years, WPC-LA programs may have limited capacity or intake processes, limiting clinical referrals. Building CBO relationships was critical to optimize appropriate social service referrals based on currently available WPC-LA services. However, relationship-building took time as CBOs sometimes questioned why overarching healthcare funding mechanisms directly funded health systems for social services referrals instead of directly existing supporting social services organizations. CBOs also asked how WPC-LA programs may overlap with existing community-based social services. In response, some collaboration team members described how they explained the rapid ramp-up phase and openly discussed the program development, resource needs, and challenges. For instance, instead of trying to “*sell WPC-LA services*” to CBOs, as one collaboration team member stated, they worked with CBOs to identify areas for collaboration, share resources, build partnerships (formally or informally), or support service needs through knowledge sharing.

Collaboration team members described a need for institutional buy-in and longitudinal infrastructure for community engagement processes, including payments or incentives for individuals with lived experience for program design or input, time for partnership building, and a need for health-system-embedded community-engaged career paths funded outside of temporary waiver funding, as the collaboration team staff were unsure of their position’s sustainability following the waiver. Longitudinal planning and collaboration infrastructure were limitations in team, partnership, and collaboration sustainability.

### Recommendations and lessons learned for other safety net systems

The Collaboration Team proposed various health system community engagement process recommendations, see Table 3. Recommendations have implications for how health systems can prepare to engage with communities effectively, be more responsive to communities, and partner to address health and social service gaps.

**Table 3:** Recommendations for community engagement in safety-net systems for social service integration

**#**	**Recommendation**	**Policy Considerations**
1	*Evaluate organizational readiness and adaptability for collaboration with the community (before engagement)*	• Conduct an internal assessment and planning process to evaluate community engagement readiness. This stage should reflect on whether any feedback received or gaps identified would be considered for systems change. The health system should explore feasible ways to adapt to potential suggestions from the community; however, if not possible, transparent explanations for decision-making should be provided. • Develop a clear purpose for community partnership/engagement for improving healthcare and social needs outcomes, which can be modified collaboratively throughout engagement processes. Concrete steps and guidance for the engagement should be defined, such as including community engagement and collaboration as an organizational value or developing a strategic plan for community engagement and collaboration. Create long-term goals to inform short-term goals (i.e., a 10-year strategic plan). • Identify power-sharing strategies, such as community positions on committees, compensation, consensus-building, or voting power, specifically focusing on those with lived or community expertise • Increase organizational readiness to discuss racism and its historical impact on communities of color; develop a framework to incorporate race and health equity into organizational practices (training, data, program planning, evaluation, etc.).
2	*Include community input and create equilateral, mutually-beneficial community partnerships*	• Incorporate community-participatory venues and processes for consensus building, bi-directional knowledge exchange, and community feedback, including planning, policy, programming, budgets, and evaluation. • The community should be supplied with funding and financial resources when providing expertise or services to improve the health systems' breadth, depth, or strategic plan. Compensate, pay, and credit the community as advisors or consultants for community time, expertise, and feedback. • Throughout the partnership, the health system should try to uplift and increase the community's exposure by highlighting their communal strengths, resources, and resiliency.
3	*Put a value on, elevate, and respect community expertise and lived experience*	• Community expertise and lived experience provide specialized cultural, socioeconomic, or community knowledge for health policy and programming, giving insight into the local community's cultural values, resources, assets, needs, and preferences. Lived experience or community expertise is critical for informing health systems to increase the quality of care by obtaining end-user feedback for the most vulnerable patients. In collaboration with social services, health systems must value the importance of community expertise and lived experience to elevate and respect the knowledge of the community they serve. • Recognize how lived experience expertise provides value for the health system by building trust, improving patient satisfaction, improving quality of care, reducing health disparities, and addressing factors within the communities' well-being, including social determinants of health.
4	*Hiring from and for the community*	• Hiring health system employees should reflect the community served to better address the community's needs, cultural unity, and behavioral norms. To develop and utilize culturally humble talent acquisition, consult the community for recruitment, hiring, and training assistance. • Consider peer support and leadership roles that emphasize lived experience as a skill and tool for patient engagement.
5	*Professional development and staff sustainability in community collaboration and engagement*	• Provide education, skill-building, career opportunities, mentorship of impacted community members, and leadership opportunities for Black, Brown, and people from affected focus communities. Invest in developing and specialization skills to build best practices for community engagement. A plan for employees for job readiness, training, and engagement or health systems skills prep should exist. Develop guidelines for recognizing autonomy, flexibility, accountability, and time investment for engagement work. • Offer staff opportunities for growth based on strengths and quality of work—the dedicated collaboration team position allowed for a richer conversation about short and long-term solutions • Increase awareness within health system leadership for best practices for authentic longitudinal community engagement and cultural humility. Train and incorporate leadership with community experience.

## Discussion

The newly implemented and dedicated collaboration team provided locally informed communication that allowed for bi-directional knowledge exchange between county services, populations with lived experience facing health disparities, direct service staff (i.e., CHWs), and community-based social services, identifying service gaps and recommendations. Through direct outreach, service assessment, and building community partnerships, collaboration team members engaged cross-county services, CBOs, and community members to facilitate health and social service networks. The collaboration team obtained critical insight through community engagement to improve social and medical service delivery for WPC-LA focus populations, resulting in health system policy recommendations, program changes, and partnerships. This novel team described building cross-sector relationships and workgroups to facilitate outreach, service assessments, and partnerships. Recommendations have implications for other health systems in creating roles for, designing, and conducting community-engaged processes for addressing health disparities, service gaps, and social needs.

The collaboration team provided recommendations for health system community engagement processes and potential institutional changes, including facilitating organizational readiness, building mutually beneficial community partnerships, valuing lived experience, and human resource strategies for incorporating community leadership and staff development in community engagement processes. Calls for institutional change for health system community partnerships described elsewhere include the need for human resource infrastructure supporting community engagement, including time for relationship building and consensus-building, decision-making power and valuing community expertise, bi-lateral problem solving for cross-sector service delivery, and data sharing, sustainable co-funding, and capacity building [1, 10, 36–48]. Additional issues in navigating cross-sector differences between health systems, social services, and community resources may include not over-medicalizing social services delivery to maintain expertise in community-based, tailored social service delivery [7, 39, 45, 49]. Evidence shows that health services staff can form synergistic community partners; however, institutional change is critical to sustaining healthcare-community partnerships [50]. To achieve this change, participatory processes, such as collective impact, may serve as a tool to improve community engagement, promote social justice, and enhance transparency in health system programming, policy, and budgeting [51–54].

Scaling social-medical models are challenging in safety-net health systems with limited resources for building and sustaining community partnerships. Early in WPC pilot implementation, many challenges to partnerships were reported not just in LA but statewide, including the need to build new infrastructure supporting effective cross-sector care coordination, sufficient time to ensure partner buy-in, consistent cross-sector communication, sharing care goals for shared clients, aligning financial incentives, and increasing investment in care service gaps [10, 55]. Community engagement processes within health systems may vary widely and lead to different outcomes [9]. LAC DHS hired the WPC-LA Collaboration Team based on lived experience, community ties, and experience with community organizing or social services. As a result, the collaboration team’s personal and professional qualifiers enabled relating to WPC-LA target populations for building trust [56] and connecting and advocating for existing community initiatives promoting social services, well-being, or health. As a result, the community engagement process in WPC-LA may differ from other California Medicaid programs or health system engagement teams (if existent). Additionally, institutional support and the ability to sustain collaboration teams through future California Medicaid funding, such as through CalAIM, will affect cross-sector communication and collaboration implications for addressing the social determinants of health [17, 57].

Limitations include a limited sample size of collaboration team members in one county health system. LAC-DHS is the nation’s second-largest municipal health system and among the most diverse, potentially enhancing the generalizability of some findings. This study focuses on the collaboration team and health system leadership perspectives, potentially differing from feedback obtained from external collaborators, such as WPC-LA CBOs, solicited separately [44]. Lastly, the study was unable to evaluate the impact of the collaboration team on health or social outcomes of WPC-LA populations, service partnerships, cross-sector or community resource connections, or trust, an area ripe for future research. Additionally, COVID-19 may have impacted partnership-building, and more research is needed to understand how the pandemic may have shifted these efforts.

## Conclusion

Integrating the coordination of social services into healthcare creates new opportunities and challenges for cross-sector community collaboration and engagement to optimize the design and promote systemic change to improve the quality of care for vulnerable Medicaid populations. A dedicated health system community engagement team allows for bi-directional knowledge exchange between county services and people with lived experience, increasing the capacity for intervening in needed services. Engaging a wide variety of stakeholders, the collaboration team supported collaboration by advocating for community expertise to inform policies, programs, and resource distribution models centered on the experiences of communities most affected by disparities. Future efforts should tailor the collaboration team role to gain community trust, feedback, and insight and to develop new collaborations to address health inequities.

## Data Availability

All data analyzed during this study are included in this published article. For any requests about the data from this study, please contact scarson@mednet.ucla.edu.

## Abbreviations

CBO: Community-based organization
LAC DPH: Los Angeles County Department of Public Health
LAC DMH: Los Angeles County Department of Mental Health
DPSS: Department of Public Social Services
LAC DHS: Los Angeles County Department of Health Services
SPA: Service Planning Area
WPC: Whole Person Care
WPC-LA: Whole Person Care-Los Angeles
